# The CBHPE Outcomes Theory of Change: An International Consensus Statement on the Mechanisms and Outcomes of Competency-Based Education in the Health Professions

**DOI:** 10.5334/pme.2553

**Published:** 2026-09-08

**Authors:** Aleda M. H. Chen, Denise H. Rhoney, Mary Ellen J. Goldhamer, Andem Ekpenyong, Susan Humphrey-Murto, Holly Caretta-Weyer, Linda Snell, Arvin Damodaran, Robert Englander, Andrew K. Hall, Soeren Huwendiek, Laura Molgaard, Anna Oswald, Daniel J. Schumacher, Fremen Chihchen Chou, Pedro Tanaka, Jason R. Frank, As members of the International Competency-based Education in the Health Professions Collaborators

**Affiliations:** 1 University of Arkansas for Medical Sciences, Little Rock, Arkansas, USA; 2 UNC Eshelman School of Pharmacy, Chapel Hill, NC, USA; 3 Division of General Internal Medicine, Massachusetts General Hospital, USA; 4 Harvard Kidney Urology Hematology Training Institute, Boston, Massachusetts, USA; 5 Department of Internal Medicine, Rush University Medical Center, Chicago, IL, USA; 6 Accreditation Council for Graduate Medical Education (ACGME), USA; 7 University of Ottawa, Canada; 8 University of Ottawa, The Ottawa Hospital, Ottawa, Canada; 9 Stanford University School of Medicine, Palo Alto, California, USA; 10 McGill University, Montreal, Canada; 11 Royal College of Physicians and Surgeons of Canada, Ottawa, Canada; 12 School of Clinical Medicine, Faculty of Medicine and Health, UNSW, Sydney, Australia; 13 Department of Medical Education, University of Illinois College of Medicine, Chicago, IL, USA; 14 Department of Emergency Medicine, University of Ottawa, Canada; 15 Royal College of Physicians and Surgeons of Canada, Canada; 16 Department for Assessment and Evaluation, Institute for Medical Education, University of Bern, Bern, Switzerland; 17 University of Minnesota College of Veterinary Medicine, St. Paul, Minnesota, USA; 18 Department of Medicine, University of Alberta, Edmonton, Canada; 19 Cincinnati Children’s Hospital Medical Center/University of Cincinnati College of Medicine in Cincinnati, Ohio, USA; 20 China Medical University Hospital, Taiwan; 21 China Medical University, Taichung, Taiwan; 22 Stanford University School of Medicine, Stanford, California, USA; 23 The Ottawa Hospital, Ottawa, Canada

## Abstract

**Introduction:**

Competency-Based Health Professions Education (CBHPE) is an outcomes-based approach to planning and conceptualizing the training of health professionals to serve population needs. Despite its uptake, ongoing debate persists regarding the assumptions underpinning CBHPE, its intended purposes, and how success should be defined and evaluated. This contributes to variable implementation and difficulty aligning core features with meaningful outcomes. To date, no published theory of change has articulated how CBHPE’s core components provide a framework for later evaluation of intended outcomes.

**Methods:**

Using Funnell and Rogers’ purposeful program theory, the authors, all members of the International Competency-based Education Health Professions Educators Collaborative (ICBHPE), developed a model theory of change. The authors conducted a broad review of CBHPE literature, supplemented by CBE frameworks from adjacent educational contexts to strengthen underdeveloped constructs in implementation and evaluation. Findings were iteratively synthesized into a program theory and refined through repeated cycles of large-group feedback, consensus building, and member checking within ICBHPE.

**Results:**

The authors developed a CBHPE Outcomes Theory of Change that articulates key assumptions, mechanisms of impact, and expected outcomes associated with CBHPE implementation. The framework specifies how van Melle’s CBHPE core components are theorized to function across phases of implementation and identifies proximal and distal outcomes that can reasonably be expected, based on intentional, deliberate program evaluation at each implementation and evaluation stage.

**Discussion:**

The CBHPE Outcomes Theory of Change offers scholars and implementers a practical guide to enhance the quality and fidelity of implementation, evaluation, and research efforts related to CBHPE worldwide. Using this guide as a framework, programs can plan, implement, and then examine whether the intended outcomes of CBHPE have been realized.

## Introduction

Competency-Based Health Professions Education (CBHPE) is an outcomes-based education approach to conceptualizing and designing curricula and educational systems for health professionals [1,2,3]. It is a collection of aligned practices intended to enhance the quality of teaching, learning, and assessment in healthcare, while refocusing efforts on ensuring every graduate is prepared to address the needs of the populations they serve [1,2,3]. Competency-based medical education (CBME) was introduced in 1978 by the World Health Organization [4]. For the last 50 years, medical educators around the world have worked to transform training using CBE principles to emphasize graduate abilities, patient and population needs, learner-centeredness, and evidence-informed educational methods. The growth of CBME has been supported by concerns that much of 20th-century medical training relied on fixed, time-based models, resulting in variable learning experiences and outcomes that emphasize knowledge acquisition over demonstrated competence and performance in practice [2,5,6,7,8,9]. CBME progressively evolved, accompanied by new approaches to curriculum design and assessment, and has been adopted (or actively explored) across many health professions in many countries [10,11,12,13,14,15,16,17,18,19,20]. (Herein, the paper will collectively refer to competency-based education in any health profession, including CBME, as CBHPE). However, some have noted gaps in the implementation of CBHPE, including persistent debate about what CBHPE entails and how its Core Components should be interpreted in practice [21,22,23,24]. Thus, our paper responds to these ongoing challenges by developing a theory-driven model that clarifies how CBHPE is intended to work, linking the Core Components, implementation processes, and expected outcomes.

In 2019, van Melle and colleagues published an expert consensus framework, the Core Components, to support a shared understanding of CBHPE and enable more consistent evaluation of implementation [3]. The framework arises from the foundational principles of CBHPE being a learner-centered, patient-focused approach to education. It specifies five Core Components: 1) outcome-based competencies (patient-focused), 2) sequenced progression (learner-centered), 3) tailored learning experiences (learner-centered), 4) competency-focused instruction (learner-centered), and 5) programmatic assessment. For each Core Component, van Melle et al described relevant underlying conceptual theories, principles, and implementation recommendations, reinforcing a transformational reform rather than a simple curricular “add-on” [3,5].

Despite the guidance provided by the Core Components framework, there remains ongoing debate about how the core components should be implemented. Much of this debate stems from heterogeneity in study purposes, designs, implementation contexts, and outcome selection rather than a lack of scholarly engagement or empirical inquiry [21,23,25,26,27]. A narrative review by Brydges and colleagues highlighted that although the evidence base for CBHPE assumptions is substantial, challenges remain, including inconsistent reporting, limited articulation of underlying assumptions, and difficulty linking implementation to outcomes [23]. While the lack of standardized or consistent reporting of these critical elements in scholarship complicates the ability to synthesize and draw large-scale conclusions across published work [28], evidence suggests that CBHPE delivers on its intended outcomes and is a valued approach to education [5,14,15,20,29,30,31,32].

Collectively, CBHPE scholarship has involved studying the wide variety of implementation strategies employed rather than a single intervention [20,32,33,34]. Studies have appropriately focused on evaluating different aspects of CBHPE implementation, including readiness for change, fidelity of implementation, enactment of core components, assessment system validity, educational outcomes, and downstream impact [35]. The focus of evaluation has shifted over time as programs have progressed through different stages of CBHPE adoption and refinement [5,35]. Thus, our paper reflects that progression by bringing together complementary contributions that examine CBHPE evaluation methods, program evolution in response to implementation challenges, explicit theories of change and outcome pathways, and reporting standards to improve coherence, interpretability, and accountability across future efforts [28,34,35,36,37,38,39,40,41,42,43,44].

While efforts to strengthen shared language, such as outcomes taxonomies and core component frameworks, have advanced the field [3,33,45], gaps remain in explicitly linking CBHPE assumptions, implementation, and expected outcomes, limiting the interpretability and comparability of findings across programs [22]. Complicating this, the degree to which a CBHPE program is implemented as intended is variable [15,21]. Misra and colleagues outline gaps in the implementation of the core components and make recommendations to address these [22]. However, programs may adopt CBHPE terminology while selectively enacting core components, making it difficult to determine whether observed outcomes reflect CBHPE as a coherent, theory-informed reform or partial and inconsistently supported implementation [3]. In the absence of explicit program theory, outcome variability risks being misinterpreted, conflating implementation failure with theory failure [46,47]. Together, these challenges underscore the need for clearer articulation of how CBHPE is expected to work, under what conditions, and with explicitly defined outcomes.

An explicit CBHPE program theory is essential to underpin and strengthen CBHPE design and implementation and to support meaningful evaluation, to advance the field. Theory, as defined by Varpio and colleagues (2020), is “a set of propositions that are logically related, expressing the relation(s) among several different constructs and propositions.” A theory can be created from a body of research, explain relationships between phenomena, and be predictive, identifying outcomes based on inputs [48]. Our paper seeks to articulate the underlying assumptions and pathways by which CBHPE is expected to produce outcomes, resulting in a program theory-driven model, the CBHPE Outcomes Theory of Change. This model further elucidates each of Van Melle’s five Core Components [3], allowing programs to identify and measure indicators of implementation progress. Importantly, we present this as a theory rather than the theory, anticipating that it will evolve through critical engagement, scholarly dialogue, empirical testing, and adaptation across diverse contexts.

## Methods: Consensus Process

### Identifying the Need for a Theory of Change

The authors of this paper are members of the International Competency-based Health Professions Educators Collaborative (ICBHPE), a consortium of approximately 90 scholars, leaders, implementers, and evaluators of CBHPE. Established in 2009, ICBHPE includes members representing six continents and diverse professional backgrounds who share a common aim: advancing CBHPE through scholarship, implementation support, and international collaboration. In 2024, ICBHPE launched a structured consensus process to identify emerging priorities in the field, culminating in the selection and refinement of 18 topics for discussion at a consensus conference hosted by Stanford University in February 2025, which was attended by more than 50 participants. As part of the Forum, the group debated the contemporary interpretation of CBHPE and reiterated a view of CBHPE as described by Van Melle’s Core Components [3].

Through iterative working-group development and facilitated deliberation, the need for a Theory of Change emerged as a top priority, leading to the formation of a 16-member author group spanning four continents, with three authors leading efforts (AC, DR, JF). Using a constructivist stance, our group of authors from multiple countries and health professions (medicine, veterinary, and pharmacy) focused on a recurring need: greater clarity regarding the assumptions, mechanisms, and intended outcomes of CBHPE. These discussions occurred from October 2024 to February 2026 through email, virtual meetings, and the in-person forum. We sought to translate the existing CBHPE literature and the authors’ collective experience into practical guidance to support program design, implementation, and evaluation.

### Program Theory and the CBHPE Outcomes Theory of Change

To structure this work, and drawing on Varpio’s (2020) definition of theory [48], we used Funnell and Rogers’ purposeful program theory (2011) as a guiding framework for the theory of change. (From here forward, the manuscript uses theory of change in the program-theory tradition described by Funnell & Rogers rather than grand theory.) This approach was selected because of CBHPE’s nature as a complex educational and organizational reform characterized by multiple components that interact and significant contextual dependencies, in which outcomes are difficult to interpret when program theory is implicit or inconsistently articulated [46]. Funnell and Rogers (2011) describe purposeful program theory as an approach that articulates a theory of action (i.e., what it does and how it is implemented) and a theory of change (i.e., the mechanisms by which the change is expected to occur). As part of this, program theory makes explicit the causal logic of a program, including mechanisms, contextual influences, and intended outcomes, to support clearer interpretation of how and why outcomes are achieved in complex interventions [46]. Further, in Funnell and Rogers’ framework, implementation can be understood as the introduction of program activities that deliberately alter baseline conditions. Then, the evaluation of the program activities focuses on the extent to which these activities produce observable changes (and the mechanisms underlying those changes) compared with what would have happened if the baseline state had remained [46].

Guided by this framework, we systematically mapped the Core Components of CBHPE [3] to an explicit theory of action and theory of change, specifying the assumed causal pathways through which these components are expected to influence teaching, learning, assessment, and competence. This process enabled us to identify key assumptions, clarify mechanisms of impact, and generate a more transparent and testable account of how and why the program implementation (i.e., CBHPE) is expected to produce observable outcomes.

### Theory of Change Development and Consensus Process

Funnell and Rogers (2011) note that program theory can be built from research evidence, knowledge, and experience [46]. Drawing on this, we used both evidence and a consensus process to incorporate all three elements.

The steps after the conference are detailed in Figure 1. Notes from the forum were disseminated, and two virtual meetings were held post-conference to discuss the idea of creating a Theory of Change, evaluate different approaches, and plan next steps. First, to create and refine this program theory, the small group writing team conducted a literature review intended to be broad, explanatory, and cross-contextual. To begin, we drew primarily from CBHPE literature. We complemented this with Competency-Based Education (CBE) literature from adjacent educational contexts (e.g., higher education and primary and secondary school education) when concepts were more fully developed outside health professions or when CBHPE evidence was sparse for specific mechanisms, enabling conditions, or implementation strategies. Several non–health professions sources were particularly useful in strengthening the guide’s operational backbone. This does not imply that CBHPE is interchangeable with K–12 or postsecondary CBE; rather, it is intended to strengthen conceptual completeness in areas where the broader CBE field has produced usable frameworks for governance, readiness, transparency, and evaluation. As an example, the Competency-Based Education Network Quality Framework [49] served as a practical quality lens for specifying implementation supports and measurement expectations. Its emphasis on institutional commitment, coherent design, transparency, and evidence-informed improvement aligned well with the guide’s focus on enabling conditions, stakeholder actions, and stage-appropriate indicators of progress [49].

**Figure 1:**
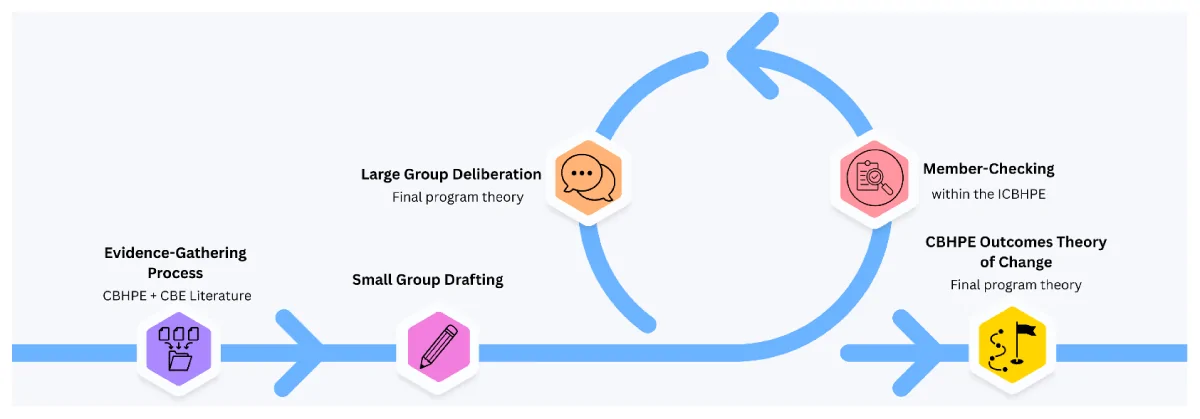
Process for Developing the Competency-Based Health Professions Education (CBHPE) Outcomes Theory of Change. This figure outlines the process for developing the CBHPE Outcomes Theory of Change. First, evidence was gathered through literature searches, and a small group drafted the Theory of Change. Then, the large group edited and deliberated drafts, alongside ICBHPE member-checking. After these iterative cycles were completed and consensus achieved, the CBHPE Outcomes Theory of Change was finalized.

We also drew on established CBE work from broader education systems to strengthen fidelity-relevant constructs that are often under-described in CBHPE reporting. The Aurora Institute/Competency Works quality principles and related “elements of quality” and system “levers” work contributed language and examples for governance coherence, educator capacity, transparent performance expectations, and equity-oriented design, all features that map directly to the guide’s barrier/enabler logic and equity lens [50]. Complementary guidance from the National Center for Education Statistics (NCES) and National Postsecondary Education Cooperative (NPEC) on competency-based initiatives reinforced the importance of credible assessment systems and stakeholder engagement, informing the guide’s inclusion of infrastructure and accountability supports as prerequisites for defensible progression decisions and interpretable outcomes [51].

As a small group writing team, three authors (AC, DR, JF) synthesized this information iteratively to develop the CBHPE Outcomes Theory of Change as a coherent program theory linking each Core Component to implementation strategies, issues the reform aims to address, assumptions and mechanisms of action, conceptual foundations, key stakeholder actions/behaviors, proximal outputs, and distal outcomes. Once drafted, the Theory of Change was shared with all authors, and refinement was achieved through repeated cycles of large-group deliberation and feedback followed by revisions by the small group writing team. Once the authors achieved consensus, member checking was conducted within ICBHPE. The paper was distributed to the entire ICBHPE for open comment, and members were invited to comment before, during, and after the forum, regardless of whether they attended. Broadly disseminating this to the ICBHPE membership enabled individuals representing the Global South and other health professions to provide feedback. Further, the Theory of Change was presented at several monthly virtual ICBHPE meetings, inviting feedback and comments from members. After these processes were completed, the CBHPE Outcomes Theory of Change was considered finalized.

## Results

The developed CBHPE Outcomes Theory of Change connects each of Van Melle’s five Core Components [3] to reform suggestions and implementation strategies. These recommendations are supported by presenting a review of the underlying assumptions, conceptual foundations, key stakeholder actions, and the expected outputs and outcomes of implementation. The CBHPE Theory of Change is an integrated set of tools designed to support implementation planning, fidelity monitoring, and the selection of stage-appropriate indicators and outcomes. Appendix 1 provides an illustrative application of the CBHPE Outcomes Theory of Change structure to the CBHPE Core Components. It is meant to serve as a practical roadmap for applying the CBHPE Theory of Change’s structure to program design, implementation monitoring, and outcomes evaluation. Table 1 provides shared definitions used across the CBHPE Outcomes Theory of Change to support consistent interpretation and application, including implementation strategies; issues to address; theory of change, including assumptions, mechanisms of action, and causal logic; stakeholder actions/behavior changes; implementation outputs/early signals; outcomes over time [proximal, intermediate, distal/impact] [3,33,45,52].

**Table 1:** Definitions of the Elements of the Program Theory Used in the CBHPE Outcomes Theory of Change.


ELEMENT OF PROGRAM THEORY	DEFINITION

**Core Component [3]**	Five foundational building blocks of contemporary Competency-Based Education (CBHPE), as described by Van Melle [3] which serves as the anchor for design and implementation (e.g., sequenced progression, programmatic assessment).

**Implementation Strategies [3,22,25,33,45,49,50,57,58,59,60]**	Specific actions, interventions, or structural changes that transform the curriculum using the core components (e.g., coaching systems, milestone mapping, feedback mechanisms)

**Drivers of Change [4,5,61]**	The underlying educational, organizational, or societal problems that the implementation strategy is trying to resolve (e.g., time-based progression, variability in learner readiness, inequitable assessment practices).

**Theory of Change [45,46,52] (Assumptions & Mechanisms)**	The rationale and assumptions about how and why these activities will lead to desired outcomes (e.g., deliberate practice improves skill acquisition; personalized feedback enhances motivation). This includes: **Assumptions:** The underlying beliefs about how and why change will happen. **Mechanisms of Action:** The processes that explain how inputs/activities lead to outcomes. **Causal Logic:** The step-by-step path from activities to impacts.

**Theoretical & Conceptual Foundations [62,63,64,65,66,67,68,69,70,71,72,73,74,75,76,77,78,79,80]**	The underlying learning science theories, conceptual frameworks, and implementation models that explain or support how the core component is intended to function in practice. These include both learner-centered theories (e.g., motivation, cognition, development) and systems-level implementation frameworks that support coherence, sustainability, and change (e.g., implementation science frameworks, change management models).

**Key Stakeholder Actions/Behavior Changes [81,82]**	Observable changes in behaviors, roles, or responsibilities of involved stakeholders (learners, faculty, administrators, clinical educators) necessary to sustain implementation (e.g., learners seeking feedback, faculty acting as coaches).

**Implementation Outputs (Early Signals) [3,15,26,27,33,45,49,50,52,53,57,69,83]**	Implementation outputs are early, observable products and signals of CBHPE implementation quality and progress. They reflect whether core components, structures, processes, and supports have been put in place and are functioning as intended, including stakeholder engagement and behavior enactment. Outputs indicate implementation fidelity and readiness for impact, and are operationalized using indicators/metrics, specific, measurable variables used to assess these early signals (e.g., adoption, fidelity, feasibility, uptake, participation). Implementation outputs are distinct from outcomes and do not represent educational, practice, or societal change.

**Outcomes Over Time (Proximal, Intermediate, Distal) [3,15,26,27,33,45,49,50,52,53,57,69,83]**	Outcomes are changes attributable to CBHPE implementation that emerge beyond early implementation signals. Outcomes are staged as proximal, intermediate, and distal/impact and align with the Hall timeline (in training → transition to practice → in practice). **Proximal (in training):** Early training-based changes indicating CBHPE is functioning as intended (e.g., learner development, assessment use, feedback quality, coaching/instructional shifts). **Intermediate (transition to practice):** Changes during transition into supervised/early practice reflecting application, consolidation, and transfer of competencies toward practice readiness. **Distal/impact (in practice):** Longer-term effects in practice and systems (sustained performance, workforce impact, patient/population outcomes, broader value).

**Barriers/Enablers [49,50,51,57,84,85,86,87,88,89,90,91]**	Factors that obstruct or facilitate change, often at the individual, cultural, or system level (e.g., resistance to change, faculty development resources, policy incentives).

**Equity Considerations [31,92,93]**	Attention to how CBE structures may promote or hinder inclusion, equity, and access for diverse learners and populations (e.g., addressing bias in assessment, ensuring fair advancement opportunities).

**Indicators/Metrics [49,50,85]**	Specific, measurable variables that can be applied across the full engine, including contextual modifiers, stakeholder behaviors, implementation inputs/outputs, and outcome tiers, using defined indicators/metrics (specific, measurable variables) to quantify implementation quality, mechanisms, and impacts across educational, professional, and societal levels


### Overarching Program Theory Model: The CBHPE Outcomes Engine and the CBHPE Theory of Change

The overarching program-theory model informing the CBHPE Theory of Change is the CBHPE Outcomes Engine (Figure 2). This theoretical model describes how CBHPE is expected to generate change when implemented with fidelity. In this model, “need for change” drivers (societal, educational, and organizational) inform implementation strategies that, when enacted through aligned stakeholder behaviors, enable the implementation of van Melle’s five CBHPE Core Components [3]. The CBHPE Outcomes Engine emphasizes that Core Components operate through interacting mechanisms specified in the Theory of Change. Each Core Component needs to be intentionally designed and implemented and is shaped by contextual modifiers, including enablers and barriers, equity considerations, and implementation/quality improvement strategies. These contextual modifiers influence the feasibility, engagement, and decision processes required for enactment and moderate the strength and consistency with which CBHPE mechanisms are activated. The model distinguishes implementation outputs from outcomes over time, representing measurable effects that emerge as CBHPE matures (proximal, intermediate, and distal/impact outcomes) [45]. Implementation outputs (early signals) reflect immediate, observable indicators that CBHPE has been initiated and is being delivered with sufficient quality to make downstream outcomes interpretable. In this model, early signals include [53]:

- Adoption: the decision or action to begin using CBHPE components (e.g., implementing programmatic assessment processes, using progression expectations, deploying coaching structures).
- Fidelity: the degree to which CBHPE is delivered as intended (i.e., alignment with the designed core components, processes, and standards), including adherence and quality of delivery.
- Feasibility: the extent to which CBHPE can be carried out successfully within the local setting, given time, workload, resources, and operational constraints.
- Sustainability: the extent to which CBHPE processes are maintained over time and become routinized (e.g., continued use after initial rollout, stable governance, ongoing calibration, and faculty development).
- Penetration: the depth and breadth of integration of CBHPE within the program and its subsystems (e.g., across courses/rotations, departments, sites, and stakeholder groups), reflecting how embedded it becomes in routine operations.
- Reach (participation rate): the proportion and representativeness of the intended stakeholders who actually engage in CBHPE processes (e.g., percent of learners completing evidence reviews, percent of faculty providing structured feedback, percent of preceptors completing assessments (i.e., evaluation completion rates)).
- Acceptability: stakeholders’ satisfaction with perceived value of, and willingness to engage in CBHPE components (often reflecting perceived burden vs benefit).
- Appropriateness: stakeholders’ perception that CBHPE is a good fit for the setting, goals, and local constraints (i.e., relevance and compatibility distinct from “liking it”).

**Figure 2:**
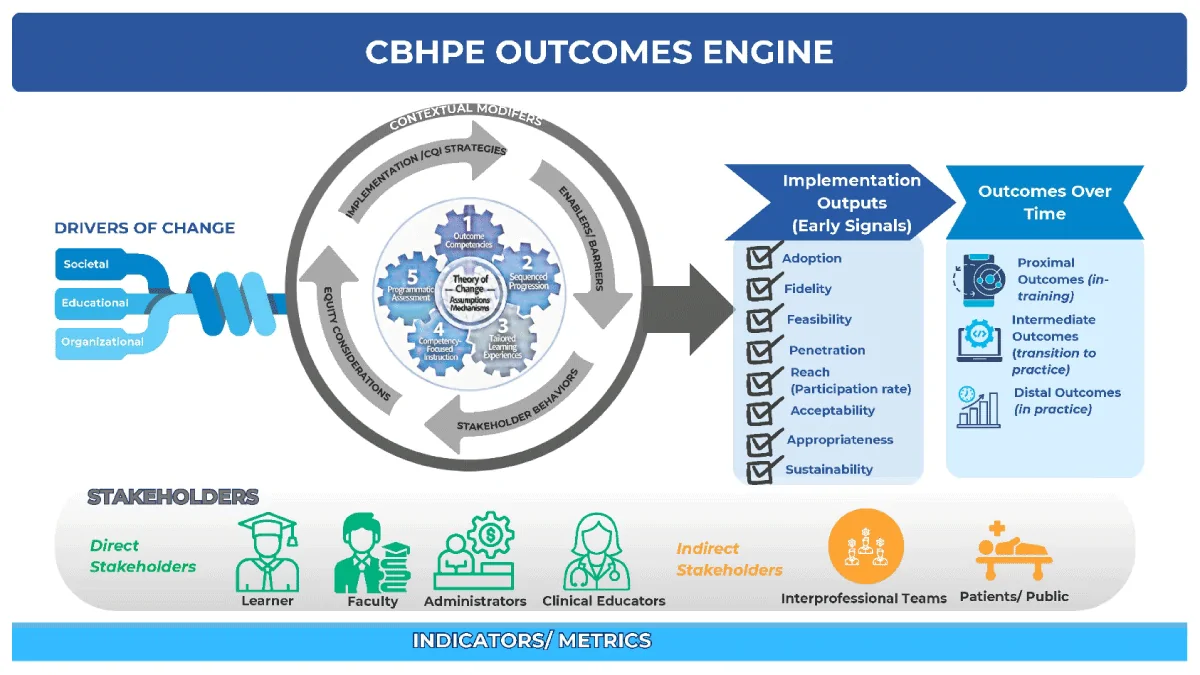
Competency-Based Health Professions Education (CBHPE) Outcomes Engine and the Theory of Change. This figure presents the CBHPE Outcomes Engine, a theory-driven model describing how competency-based health professions education (CBHPE) translates a need for change (societal, educational, and organizational) into measurable implementation signals and outcomes over time. The model emphasizes that outcomes are not assumed; they emerge when stakeholder behaviors activate the mechanisms specified in the theory of change within a given implementation context. At the center, the theory of change represents the key assumptions and causal mechanisms linking behavior change to outcomes. The theory of change is positioned centrally to indicate that aligned behaviors, enacted by direct stakeholders, provide a framework of how the five core components of CBHPE (outcome competencies, sequenced progression, tailored learning experiences, competency-focused instruction, and programmatic assessment) produce outcomes. However, these outcomes must be cultivated through rigorous planning, implementation, and examination of outcomes. Direct stakeholders (e.g., learners, faculty, administrators, and clinical educators/workplace educators) are those whose actions and behaviors are intentionally targeted by, and integrated into, CBHPE design and implementation, and who constitute the primary levers through which CBHPE functions. Contextual modifiers surrounding the core components represent system conditions that shape whether and how effectively CBHPE mechanisms are activated and sustained. These include enablers and barriers, equity considerations, and implementation/quality improvement strategies. Contextual modifiers influence stakeholder behaviors (e.g., feasibility, engagement, decision processes) and moderate the strength and consistency of implementation, thereby shaping the interpretability of outcomes. To the right, implementation outputs (early signals) reflect immediate, observable indicators that CBHPE has been initiated and is being delivered with sufficient quality to make downstream outcomes interpretable. In this model, early signals include: adoption, fidelity, feasibility, penetration, reach, acceptability, appropriateness, and sustainability [53]. These implementation outputs are operationalized using indicators/metrics, specific, measurable variables used to quantify early signals (e.g., evaluation completion rates, observation counts, inter-rater calibration results, time-to-feedback, coverage across sites, resource utilization). These outputs are distinct from outcomes over time, which represent effects that emerge as CBHPE matures. Outcomes are staged over time as proximal (in training), intermediate (transition to practice), and distal/impact (in practice) outcomes, aligning the outcome tiers with the Hall taxonomy timeline (training → transition to practice → practice). This sequencing aligns with CBME outcomes literature in which early implementation outputs establish interpretability and precede longer-term outcomes [3,33,45,52]. The stakeholder band distinguishes between direct and indirect stakeholders. Indirect stakeholders (e.g., interprofessional teams and patients/public [54]) are influenced by and may influence CBHPE through system-level interactions and downstream effects, but their behaviors are not necessarily the primary targets specified in the CBHPE intervention. Together, the model provides a coherent framework for understanding how behavior-driven implementation of CBHPE, shaped by contextual modifiers, generates early implementation signals and progressively meaningful outcomes across educational, professional, and societal levels. Finally, the Indicators/Metrics band denotes that measurement can be applied across the full engine, including contextual modifiers, stakeholder behaviors, implementation outputs, and outcome tiers, using defined indicators/metrics (specific, measurable variables) to quantify implementation quality, mechanisms, and impacts across educational, professional, and societal levels.

These occur through intentional thought, planning, and deliberate design. Collectively, the components of the CBHPE Outcomes Engine provide a coherent framework for interpreting CBHPE findings by linking behavior-driven implementation, context, and early implementation signals to evaluation of progressively meaningful outcomes across educational, professional, and societal levels. All these factors/influences together strengthen the implementation fidelity of CBHPE, setting the stage for robust outcome evaluation and advancement of CBHPE.

### Practical Application: How to Use the CBHPE Outcomes Theory of Change Model

Perhaps the most practical approach to bring the CBHPE Outcomes Theory of Change to life is to consider its applications for key stakeholders (Box 1).

**Box 1 Practical Application: How Different Constituencies Can Use the CBHPE Outcomes Theory of Change**

**Figure:**
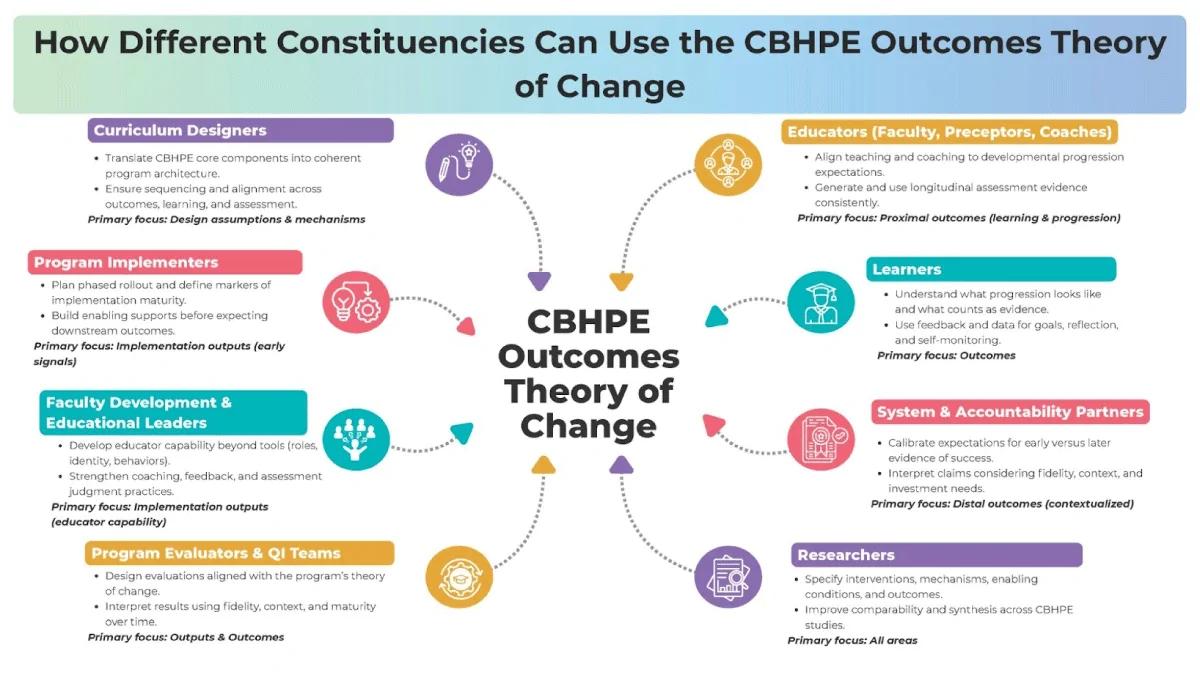

The CBHPE Outcomes Theory of Change is intended as a working model for implementation and evaluation, not simply a conceptual description. It helps teams make explicit: (1) what must be in place for CBHPE to function, (2) which mechanisms are expected to produce change, (3) which enabling conditions shape fidelity and feasibility, and (4) which indicators should be expected early versus later as implementation matures. The applications in the text illustrate how different constituencies can use the model to guide design, implementation, evaluation, and scholarship.

In sum, the CBHPE outcomes theory of change functions as a shared interpretive guide that aligns expectations, measurement, and improvement across roles, supporting more coherent implementation, more interpretable evaluation, and more comparable scholarship.

Curriculum designers can use the theory of change to translate CBHPE principles into a coherent program architecture. The Core Component maps (Appendix 1) help clarify which components should be present, how they connect, and what should be prioritized during design (e.g., sequencing developmental markers, aligning learning experiences with competencies, and ensuring assessment plans can document progression). Used as a design check, the CBHPE Theory of Change can surface missing components, unrealistic assumptions, or misalignment between intended outcomes and available structures, consistent with alignment principles and the broader CBE quality literature that emphasizes coherence and transparency as prerequisites for effectiveness.

For program implementers, the CBHPE Outcomes Theory of Change can be used to plan rollout and monitor implementation quality over time. By making enabling conditions and stakeholder actions explicit, the theory of change helps teams anticipate predictable barriers (e.g., workflow constraints, role confusion, insufficient capacity for observation/coaching) and target supports (e.g., educator development pathways, protected time, fit-for-purpose assessment tools, usable data systems). This mirrors broader CBE emphasis on “system supports” and the complex-intervention insight that implementation quality often precedes outcome change.

Faculty development leaders can use the theory of change to design training that goes beyond tool use and supports the behavioral and identity shifts required for CBHPE (e.g., observation and feedback, high-quality assessment judgments, growth mindset, development view of competence, coaching over time). By linking core CBHPE components to underlying mechanisms and expected outcomes, the theory of change helps define what “competence” looks like for educators implementing CBHPE; for example, the ability to make defensible entrustment decisions based on multiple observations; to provide specific, actionable feedback aligned with outcome competencies; and to use assessment data formatively to guide learner progression over time. In this way, educator competence is conceptualized not as procedural compliance with CBHPE tools, but as skilled implementation of assessment and feedback practices shown to drive learner improvement.

For program evaluators, the CBHPE Outcomes Theory of Change can support the construction of evaluation plans aligned to a given program’s theory of change. Rather than measuring only distal outcomes, the theory of change supports a balanced portfolio of indicators across implementation, outputs, and outcomes. This includes fidelity and implementation quality so results can be interpreted appropriately and compared more meaningfully across settings. This approach is consistent with process evaluation guidance for complex interventions. It supports the critical distinction between “CBHPE does not work” and “CBHPE was not implemented with necessary conditions, alignment, or elements.”

Frontline educators can use the CBHPE Outcomes Theory of Change to align day-to-day teaching, coaching, and feedback with developmental progression. It clarifies what learners are working toward, how learning experiences support progression, and how assessment information should be generated and used. This alignment strengthens the interpretability of assessment signals and supports consistent coaching expectations across learning environments.

CBHPE is a learner-centered approach to education. Thus, it is essential for learners to understand what CBHPE aims to accomplish, how it can support their development, and what progression should look like in practice. The CBHPE Outcomes Theory of Change can support this by clarifying how competencies, milestones, learning plans, and assessment data align, thereby enhancing transparency. Thus, enabling learners to engage more intentionally in goal setting, self-monitoring, feedback-seeking, and reflection – all processes consistent with formative assessment theory and mastery learning principles that are central to CBHPE.

System and accountability partners, including accrediting and regulatory bodies, health systems, and clinical partners, future employers, professional organizations, funders, patients, and the public [54], can use the CBHPE Outcomes Theory of Change to calibrate expectations about which evidence should emerge early versus later, and which investments are necessary for success. The CBHPE Theory of Change offers a structured way to interpret performance claims, avoid over-reliance on single metrics, and account for fidelity and context. It can also support accountability and improvement conversations by making the infrastructure and system supports (e.g., workforce development, data systems, practice environments) required to achieve intended outcomes transparent, thus reflecting the emphasis on institutional commitment and equity-oriented design in the broader CBE quality literature.

Finally, researchers can use the CBHPE Outcomes Theory of Change as a shared conceptual framework to improve comparability across studies and settings. The CBHPE Outcomes Theory of Change supports clearer specification of interventions (which Core Components [3], which mechanisms, which enabling conditions), better aligned outcome selection, and more interpretable results. Advancing these goals is consistent with calls for shared CBHPE outcome language and stronger reporting of implementation quality, mechanisms, and context.

## Discussion

The development of the CBHPE Outcomes Theory of Change addresses a persistent interpretive challenge in CBHPE: as CBHPE outcomes reporting grows, it remains difficult to draw meaningful conclusions when program theory is implicit, implementation is described variably, and outcomes are selected without a clear linkage to mechanisms or to the stage of implementation. Program theory approaches argue that complex reforms should be evaluated not only by whether outcomes change but by how implementation unfolds, which mechanisms are activated, and how context shapes effects [46,55]. In that spirit, the CBHPE Outcomes Theory of Change makes explicit how the five Core Components [3] link to assumptions, enabling conditions, mechanisms, implementation outputs (early signal), and outcomes over time that can be monitored and interpreted across stages of implementation. However, the authors recognize that this should be a living document that is discussed and refined over time as well as adapted based on context, resources, and profession. Adopters and implementers of CBHPE should test the Theory of Change, suggest modifications, and address emergent phenomena that are a natural part of complex systems [56], and evolve the theory to meet needs. For example, these evolutions could include refining outcome sets to prioritize those most predictive of successful implementation and sustainability. It is not intended to be a rigid or concrete theory; instead, the goal is to enhance the science surrounding CBHPE.

A key contribution of the CBHPE Outcomes Theory of Change is that it reframes the longstanding debate about CBHPE away from the question of whether CBHPE “works” as a single intervention, and toward more actionable questions: which Core Components were enacted, through what mechanisms, under which enabling conditions, and what forms of evidence should reasonably be expected at different phases of implementation? This aligns with concerns raised in the literature that core assumptions are often carried forward without consistent articulation of evidentiary support or clear specification of what would count as confirming or disconfirming evidence [23,26,27]. As Cooper and Holmboe recently highlighted, CBHPE is a complex social systems intervention whose success depends less on the adoption of discrete tools and more on the coherent integration of competency frameworks, programmatic assessment, faculty development, governance structures, and longitudinal learning environments [5]. By emphasizing mechanisms, roles, and phases of implementation, the CBHPE Outcomes Theory of Change offers a practical scaffold for operationalizing these principles in CBHPE research, clarifying which outcomes are plausible to observe, when, and under what conditions, thereby supporting the generation of comparable evidence.

The CBHPE Outcomes Theory of Change is also intended to clarify questions of implementation fidelity and variation. CBHPE is frequently positioned as a pathway to reduce unwarranted variation in graduate outcomes by strengthening alignment among competencies, instruction, assessment, and progression decisions. However, as Holmboe and Kogan argue, unwarranted variation can also emerge when standards, assessment practices, and implementation support are inconsistent, particularly when programs adopt CBHPE language while enacting only partial elements of the model [21]. By foregrounding enabling conditions, stakeholder actions, and proximal outputs that indicate whether mechanisms are actually being activated, the guide offers a structure for interpreting variability more defensibly, distinguishing warranted contextual adaptation from partial or misaligned implementation. Further, using cross-sector sources (i.e., the broader CBE literature) strengthened the guide’s ability to operationalize “high-quality CBHPE” in practical terms by clarifying which enabling conditions should be in place, what implementation quality can look like, and what forms of evidence are reasonable to expect as programs mature.

Building on prior competency-based medical education logic model and outcomes taxonomy work [45,52], this paper extends prior logic model approaches in two ways. First, we make the underlying theory of change (how and why a change is expected to occur) explicit by articulating the assumptions, mechanisms of action, and stakeholder behaviors through which implementation of the Core Components is expected to generate outcomes over time. Second, we operationalize these pathways into practical outcome maps and candidate indicators to support consistent outcome selection, measurement, and reporting across diverse CBHPE contexts. This extension is important because CBHPE functions as a complex, behavior-dependent educational reform. Without explicit causal pathways and conditions, variability in outcomes can be difficult to interpret and may be misattributed to theoretical failure rather than implementation or contextual constraints [6]. Outcome variability reflects differences in context, implementation conditions, and activation of underlying mechanisms. Although this work does not undertake a realist evaluation, clarifying assumptions and causal pathways supports a more rigorous interpretation of outcomes and creates opportunities for future realist-informed evaluations and reporting.

### Limitations

This theory of change was developed through an international consensus process and an evidence-gathering approach intended to be broad and explanatory rather than systematic or exhaustive. It should therefore be interpreted as a consensus-derived program theory that integrates scholarship and collective expertise, rather than as a graded evidence synthesis or an estimate of intervention effects. In addition, the CBHPE Outcomes Theory of Change is designed for cross-profession and cross-country usability; this breadth increases transferability but may reduce operational specificity for any single program, making local adaptation essential. While the Collaborative has members from six continents and many countries, the authors will inevitably reflect their lived experiences and perspectives. The authors hold leadership roles in academic institutions, accreditation and assessment activities, and health professions education organizations, and several have contributed to the development, implementation, evaluation, and scholarship of CBME/CBHPE. However, the Collaborative and the author team for this paper have a heavy Global North representation, with less Global South representation. Therefore, while our Global North experiences inform our perspective, they also risk Global North bias, which may affect our ability to fully conceptualize meaningful applicability in Global South contexts. We hope to engage in scholarly discourse with the Global South to refine further and adapt this theory. While we view CBHPE as an evolving response to limitations of traditional training models, we recognize that our familiarity with its concepts and infrastructure may influence assumptions about reporting. To mitigate this, we grounded the guidelines in established implementation science, program theory, and evaluation frameworks and engaged in iterative, multidisciplinary, and international dialogue to support interpretability across contexts and stages of implementation.

### Future Directions

The CBHPE Outcomes Theory of Change provides a platform for a more cumulative science of CBHPE by making theory explicit and testable. Next steps include empirical evaluation of its utility, identification of indicators that remain sensitive to local constraints, and assessment of whether the guide improves reporting quality. We take the next steps in this direction in a companion paper about using the Theory of Change to create CBHPE implementation reporting guidelines [28].

Future work should also explicitly test equity implications, including how assessment and progression practices may mitigate or amplify bias, and which enabling conditions are necessary to support fair, trustworthy decisions over time. Further, as mentioned previously, the Theory of Change should be revisited and modified at regular intervals to refine and address emergent phenomena over time.

## Conclusion

As CBHPE continues to expand globally, the field needs a clearer, shared articulation of how CBHPE is intended to function, what must be in place for the Core Components to work as a system, which mechanisms are expected to drive change, and what evidence should reasonably be expected across phases of implementation. Complex interventions that represent paradigm shifts often are messy but can be impactful [6]. Thus, the CBHPE Outcomes Theory of Change offers a consensus-informed, theory-based map that links the five Core Components to enabling conditions, stakeholder actions, proximal outputs, and longer-term outcomes, providing a practical foundation for more consistent implementation and more interpretable evaluation. By making CBHPE’s program theory explicit, the guide supports the further development of CBHPE, enabling programs, evaluators, and researchers to assess fidelity, select stage-appropriate indicators, compare findings across settings, and refine CBHPE toward its intended educational and societal impacts.

## Additional File

The additional file for this article can be found as follows:

10.5334/pme.2553.s1 Appendix 1. The CBHPE Outcomes Theory of Change for CBHPE Core Components.
