# Supplementary material for: The CBHPE Outcomes Theory of Change: An International Consensus Statement on the Mechanisms and Outcomes of Competency-Based Education in the Health Professions
Source: Perspect Med Educ. 2026 Sep 8;15(1):732–47. doi: 10.5334/pme.2553 (PMC13629535; doi:10.5334/pme.2553)
Supplement: Supplementary file 1 [file pme-15-1-2553-s1.pdf]

APPENDIX I. The CBHPE Outcomes Theory of Change for CBHPE Core Components

APPENDIX GUIDE

Purpose and relationship to the main text.

The following appendices provide illustrative applications of the CBHPE Outcomes Engine (Figure 1) across the five core components of competency-based health professions education described by van Melle [3]. Table 1 defines the program-theory elements that structure the Outcomes Engine; these appendices show how those elements can be operationalized at the core-component level, including example indicators and metrics.

How to read the appendix tables.

Each core component table follows a consistent sequence that mirrors Figure 1 and Table 1: (1) the drivers of change motivating implementation, (2) implementation strategies and the underlying theory of change (assumptions and mechanisms), (3) theoretical/conceptual foundations, (4) key stakeholder actions/behavior changes, (5) implementation outputs (early signals) with potential CQI indicators/metrics, (6) outcomes staged over time as proximal (in training), intermediate (transition to practice), and distal/impact (in practice), and (7) contextual moderators (barriers/enablers and equity considerations). This common structure supports sense-making and cross-component comparison.

Interpretive guidance.

The examples and potential indicators/metrics are intentionally selective (not exhaustive) and are offered for illustration, not as prescriptive requirements or a comprehensive implementation manual. Because CBHPE is context-sensitive, programs should interpret and adapt these examples to their local environment (e.g., program type, learner population, practice settings, regulatory requirements, culture, and available resources/data systems). They may need to develop locally tailored guidance that specifies operational definitions, thresholds, and feasible measurement plans. “Implementation outputs” reflect early, observable signals that a core component is in place and functioning (e.g., adoption, uptake, fidelity/consistency, feasibility, engagement), whereas “outcomes over time” represent effects that may emerge as implementation outputs and stakeholder behaviors are sustained.

CORE COMPONENT 1: OUTCOME COMPETENCIES

Core Component 1: Outcome Competencies Elements	Description and Examples	Example Potential Indicators / Metrics
	Competencies required for practice are clearly articulated, measurable, and transparent to all stakeholders. These competencies serve as the foundation for curriculum design, instructional alignment, and assessment practices [3].	• competency framework exists (Y/N)<br>• % competencies with behavioral descriptors/anchors; presence of proficiency levels/milestones (Y/N)<br>• version date + governance owner listed (Y/N)<br>• stakeholder awareness score (e.g., % correct on short "find/define" quiz)<br>• access/usage analytics (# visits/downloads per month)
**Implementation Strategies [3, 22, 25, 33, 45, 49, 50, 57-60]**	• Develop/adopt a competency framework (and EPAs/milestones where applicable) with shared definitions and performance descriptors.<br>• Map competencies to courses/rotations and assessment touchpoints (curriculum map).<br>• Embed competency language into assessment tools and feedback templates (competency/ EPA tags).<br>• Build governance/version control (review cadence, update process).<br>• Faculty/preceptor development and rater calibration using shared anchors.	• % courses/rotations mapped<br>• % assessments with competency tags<br>• # calibration sessions/term<br>• % faculty/preceptors completing calibration<br>• governance review completed on schedule (Y/N)<br>• mean time from requested change → approved update (days)
**Drivers of Change [4, 5, 61]**	• Variable learner preparedness for the next phase of education/training<br>• Variable and/or implicit expectations for competence across courses, sites, and assessors, leading to inconsistent judgments and progression decisions.<br>• Misalignment between curriculum, assessment, feedback language and real-world performance.<br>• Limited transparency of performance standards for learners, reducing goal-setting, feedback seeking, and self-regulation.	• between-site/rater variance for comparable learners (e.g., variance components/ICC)<br>• calibration concordance on benchmark cases (% within 1 level)<br>• % assessments lacking explicit competency linkage<br>• % competencies with no mapped teaching or assessment touchpoint<br>• learner "clarity of expectations" score (1–5) and % unable to identify expected performance level for priority competencies<br>• subgroup differences in ratings/entrustment (with adjustment where feasible)<br>• distribution of high-value learning experiences/observations/coaching encounters by subgroup and site
**Theory of Change [45, 46, 52] (Assumptions & Mechanisms)**	**Assumption:** transparent, measurable competencies create a shared standard that enables coherent assessment and progression decisions.<br><br>**Mechanisms:** shared mental model → aligned assessment language/tools → higher-quality competency-referenced feedback → improved learner self-regulation/goal setting → more consistent judgments and defensible progression decisions → improved readiness at transition and in practice.	• calibration concordance on benchmark cases (% within 1 level)<br>• % feedback comments explicitly linked to competencies<br>• learner learning plans mapped to competencies (%)<br>• % progression decisions referencing competency evidence trail
**Theoretical & Conceptual Foundations [62-80]**	• Outcomes-based education<br>• Criterion-referenced standards<br>• Validity/interpretation argument logic (construct → assessment → decision)<br>• Shared mental models and rater cognition<br>• Transparency and fairness as prerequisites for interpretability	• scoring anchors/rubrics present for priority competencies (Y/N)<br>• periodic review of competency language for clarity/bias (Y/N)<br>• narrative feedback quality score (rubric-based)<br>• documentation completeness for committee decisions (% with required elements).

**Key Stakeholder Actions / Behavior Changes [81, 82]**	**Learners**<br>• Recognize that the outcomes define the graduation requirements and that this drives all other aspects of the program<br>• Use competencies for goal setting, feedback seeking, and self-monitoring.<br>• Interpret feedback explicitly in relation to competencies.<br>• Maintain a competency-mapped learning plan and update it with performance evidence over time<br>**Faculty**<br>• Recognize that the outcomes define the graduation requirements and that this drives all other aspects of the program<br>• Design instruction/assessment using shared competency language.<br>• Provide feedback that explicitly references competencies.<br>**Preceptors/workplace educators**<br>• Recognize that the outcomes define the graduation requirements and that this drives all other aspects of the program<br>• Reinforce competencies in practice settings.<br>• Assess and coach using shared expectations.<br>• Document competency-linked observations/entrustment evidence<br>**Administrators/program leaders**<br>• Maintain governance and version control.<br>• Ensure transparency and accessibility across environments.<br>• Provide infrastructure and protected time for stakeholder training, calibration, and use of competency-linked tools.	**Learners:**<br>• % learners with competency-mapped learning plan (ILP) updated each term with linked evidence (e.g., feedback/assessments)<br>• # feedback-seeking events logged/term<br>• self-assessment–supervisor alignment (absolute difference score)<br>**Faculty/preceptors/workplace educators:**<br>• % completing calibration<br>• % observed assessments/feedback entries documented with competency/entrustment linkage<br>• # competency-tagged feedback entries per learner per month<br>• rater drift index over time.<br>**Administrators/program leaders**<br>• # governance meetings/actions logged<br>• % sites with access to tools/dashboards<br>• turnaround time for updates (days)<br>• frequency of monitoring reports reviewed (e.g., quarterly)<br>• % CQI/equity action items closed-loop
**Implementation Outputs (Early Signals) [3, 15, 26, 27, 33, 45, 49, 50, 52, 53, 57, 69, 83]**	• A finalized set of program competencies (and corresponding EPAs/milestones, as applicable) is published, accessible, and version-controlled (i.e., dated, owned, and updated through a defined review process).<br>• Competencies/EPAs are embedded in key learning and assessment touchpoints across settings (e.g., rotation goals, evaluation forms, workplace-based assessment tools, entrustment forms, feedback templates).<br>• Faculty, supervisors/preceptors, and learners can apply the competency language consistently (demonstrated through calibration activities, shared scoring anchors, or periodic alignment checks).<br>• A program-level competency-to-curriculum/rotation map exists showing where competencies are taught/coached and where evidence is collected (at least at a high level).	• Framework posted and version dated (Y/N)<br>• % courses/rotations with competency-tagged objectives/forms<br>• % assessment tools with embedded competency/ EPA tags<br>• tool usage (# accesses/logins/month)<br>• calibration completion rate (%)<br>• calibration concordance on benchmark cases<br>• between-site variance in ratings<br>• curriculum/rotation map completed and current (Y/N, last updated date)
**Outcomes Over Time (Proximal, Intermediate, Distal) [3, 15, 26, 27, 33, 45, 49, 50, 52, 53, 57, 69, 83]**	**Proximal (in training):**<br>• improved perceived clarity of performance expectations/standards<br>• higher-quality competency-referenced feedback<br>• increased learner self-regulated learning behaviors aligned to competencies (goal-setting, monitoring, feedback seeking)<br>**Intermediate (transition to practice):**<br>• improved continuity of expectations across settings<br>• fewer late-stage performance "surprises" at transition<br>• smoother onboarding into supervised/early practice roles.<br>**Distal (in practice):**<br>• sustained competent performance in independent practice and maintenance of professional development behaviors consistent with readiness for unsupervised practice	**Proximal (in training):**<br>• timing of detection of learner in difficulty (i.e., earlier detection)<br>• % learners/faculty reporting clear understanding of performance expectations (survey item/scale; e.g., agreement 1–5)<br>• feedback quality score using a structured rating approach (e.g., specificity/actionability rubric)<br>• % feedback comments explicitly linked to a competency/ EPA (tagged or coded)<br>• % learners with a competency-mapped learning plan/ILP updated at least once per term with linked evidence (feedback/assessment artifacts)<br>• frequency of documented feedback-seeking events per learner per term (log or portfolio

		entries)<br>**Intermediate (transition to practice):**<br>• supervisor readiness rating at 4–8 weeks (1–5)<br>• # early performance concerns flagged in first 8 weeks<br>• remediation/extra supervision rates during transition<br>• reduction in between-site variance for comparable learners<br>**Distal (in practice):**<br>• % at expected supervision/entrustment level at 6–12 months<br>• rate of documented performance concerns/complaints in first year of practice<br>• practice-pattern alignment/adherence to care pathways (where measurable)<br>• CPD/self-directed learning engagement (portfolio activity or CME participation rate)
**Barriers / Enablers [49-51, 57, 84-91]**	**Common barriers**<br>• Lack of shared language across departments or practice sites.<br>• Limited familiarity with outcome-based design and competency-referenced assessment.<br>• Capacity constraints (faculty/preceptor availability and competing mission demands) that limit administrative support, protected time, training, and data review.<br>**Common enablers**<br>• Leadership support and clear ownership/governance.<br>• Faculty, preceptor, and learner development focused on competency use (shared anchors, calibration).<br>• Accessible curricular mapping, documentation, and data tools to support consistent use and interpretation.	• % faculty/preceptors completing training/calibration (by site)<br>• perceived preparedness for outcome-based design (survey 1–5)<br>• protected time available for assessment/coaching activities (hours/term or survey adequacy 1–5)<br>• faculty-to-learner/preceptor-to-learner ratios or workload indices<br>• frequency of calibration/data review meetings (#/term)<br>• % on-time assessment submissions; median time-to-complete assessments<br>• tool/dashboard access and usage (# logins/month)<br>• CQI closed-loop completion rate (% action items completed)
**Equity Considerations [31, 92, 93]**	Ensure competency language, assessment practices, and access to learning and assessment opportunities are equitable and bias-aware, so all learners have a fair chance to achieve the outcome competencies.	• distribution of key learning experiences/exposures by learner subgroup and site (case mix/opportunity index)<br>• number of direct observations/assessments per learner by subgroup (mean and variance)<br>• coaching/feedback frequency per learner by subgroup<br>• time-to-entrustment or time-to-achieve expected supervision level by subgroup; differential ratings/entrustment decisions by subgroup (with adjustment where feasible)<br>• narrative feedback bias indicators (language audits)<br>• appeal/remediation rates by subgroup<br>• proportion of equity-flagged findings that trigger CQI actions and closed-loop completion (%)

CORE COMPONENT 2: SEQUENCED PROGRESSIVELY

**Core Component 2: Sequenced Progressively Elements**	**Description and Examples**	**Example Potential Indicators / Metrics**
	Sequenced progression means learners advance through a developmental continuum toward outcome competencies, with increasing complexity, independence, and expectations over time (often operationalized via milestones/levels, entrustment supervision scales, or staged performance expectations) [3].	• progression framework exists (Y/N)<br>• # competencies with defined developmental levels<br>• presence of expected thresholds at key timepoints (Y/N)<br>• % stakeholders who can correctly describe progression expectations (survey/quiz)
**Implementation Strategies [3, 22, 25, 33, 45, 49, 50, 57-60]**	• Define progression levels/milestones (or entrustment/supervision levels) for priority competencies/EPAs with clear descriptors and thresholds.<br>• Set timepoints/checkpoints (e.g., end of term/rotation/year) for expected progress and review.<br>• Align learning experiences and assessments to progression levels (blueprinting).<br>• Establish decision rules for advancement, support plans, and remediation pathways.<br>• Implement longitudinal tracking (dashboards/portfolios) to visualize trajectory and identify learners off-trajectory.	• % competencies/EPAs with progression descriptors and thresholds<br>• # checkpoints per year; % assessments mapped to progression levels<br>• presence of advancement decision rules (Y/N)<br>• dashboard/portfolio adoption (# users/logins/month)
**Drivers of Change [4, 5, 61]**	• Time-based progression allows advancement despite variable readiness.<br>• Inconsistent expectations for “increasing independence” across settings and assessors.<br>• Late identification of learners off-trajectory (support comes too late).<br>• Progression decisions based on fragmented data rather than trajectories.	• proportion of progression decisions made without longitudinal evidence (audit)<br>• frequency of “late surprises” (concerns first documented near transition points)<br>• between-site variance in supervision/entrustment expectations<br>• % learners requiring urgent remediation late in training/at transition
**Theory of Change [45, 46, 52] (Assumptions & Mechanisms)**	**Assumption:** making progression expectations explicit and developmental enables targeted learning, coaching, and defensible advancement decisions.<br><br>**Mechanisms:** clear staged expectations → aligned teaching/coaching and assessment → earlier detection of off-trajectory learners → targeted supports → improved trajectory consistency and readiness for transition/practice.	• earlier time-to-first meaningful feedback/flag for off-trajectory learners<br>• proportion of learners with documented targeted support plans when off-trajectory<br>• improved concordance between observed performance and assigned progression level (calibration)
**Theoretical & Conceptual Foundations [62-80]**	• Mastery learning and deliberate practice<br>• developmental trajectories<br>• self-regulated learning<br>• competence as a progression rather than a point-in-time state<br>• entrustment/supervision logic for workplace performance<br>• validity/interpretation arguments for progression decisions	• presence of milestone/entrustment anchors (Y/N)<br>• rater calibration concordance on level assignments<br>• documentation quality of progression decisions (evidence-to-decision audit)
**Key Stakeholder Actions / Behavior Changes [81, 82]**	**Learners**<br>• Use progression expectations to plan deliberate practice toward the next level<br>• Monitor their trajectory over time using assessment evidence<br>• Seek feedback targeted to next-level behaviors<br>• Update learning plans with progression evidence and adjust goals accordingly<br>**Faculty / Preceptors**<br>• Coach learners toward “next level” behaviors using shared anchor<br>• Assign progression/entrustment levels consistently<br>• Flag concerns early with actionable support plans<br>• Document progression evidence and rationale to support longitudinal interpretation	• learner ILP goals explicitly tied to next-level expectations (%)<br>• % learners with ILP updated at each checkpoint<br>• # targeted feedback items per learner per term linked to progression level<br>• % faculty/preceptors completing calibration<br>• calibration concordance on progression level assignments<br>• time from concern identification → support plan initiation (days)

	and decisions<br>**Administrators / Program Leaders**<br>• Review aggregate learner progression data at defined checkpoints<br>• Apply shared decision rules for advancement and support<br>• Initiate supports or remediation early for learners off-trajectory<br>• Ensure consistency of progression expectations and decisions across sites<br>• Monitor progression patterns and variability to identify unwarranted variation and guide continuous quality improvement	• % checkpoint reviews completed on schedule; completeness of evidence trail for advancement decisions (% with required elements)<br>• between-site variance in progression or supervision/entrustment assignments for comparable learners
**Implementation Outputs (Early Signals) [3, 15, 26, 27, 33, 45, 49, 50, 52, 53, 57, 69, 83]**	Early, observable signals that sequenced progression is implemented and functioning:<br>• developmental expectations are defined across stages (e.g., milestones or expected levels on supervision/entrustment scales) and progression criteria are documented and communicated<br>• assessments generate progression level/entrustment data<br>• longitudinal tracking of learner trajectories is implemented (dashboards/portfolios)<br>• checkpoints occur and decision rules are applied<br>• early support pathways are activated for learners off-trajectory	**Adoption/Reach:**<br>• % programs/courses/rotations using progression scales<br>• % stakeholders trained; dashboard/portfolio usage (# logins/month)<br>**Uptake:**<br>• % learners with progression level recorded at each checkpoint<br>• % assessments including a progression level/entrustment rating<br>**Fidelity/Consistency:**<br>• calibration concordance on level assignments<br>• between-site variance in level assignments for comparable learners<br>• % checkpoint reviews completed on schedule<br>• % off-trajectory learners with documented support plans<br>**Feasibility:**<br>• median time-to-complete progression ratings<br>• completion rates<br>• acceptability score<br>**Participation:**<br>• attendance rate at checkpoint review meetings<br>• % sites participating in calibration/alignment checks.
**Outcomes Over Time (Proximal, Intermediate, Distal) [3, 15, 26, 27, 33, 45, 49, 50, 52, 53, 57, 69, 83]**	**Proximal (in training):**<br>• improved perceived clarity of progression expectations (current level and “next level” behaviors)<br>• higher-quality progression-anchored coaching and feedback<br>• increased learner self-regulated learning behaviors aligned to progression levels (goal-setting, monitoring, feedback seeking)<br>**Intermediate (transition to practice):**<br>• improved continuity of progression expectations across settings<br>• fewer late-stage performance “surprises” at transition<br>• smoother onboarding with supervision aligned to readiness in supervised/early practice roles<br>**Distal (in practice):**<br>• more consistent early-career performance and appropriate independence<br>• stronger reliability of readiness judgments across cohorts	**Proximal (in training):**<br>• % learners/faculty reporting clear understanding of current/next progression expectations (survey item/scale; e.g., agreement 1–5)<br>• feedback quality score using a structured rating approach (e.g., specificity/actionability and explicit linkage to next-level anchors)<br>• % feedback comments explicitly linked to a progression level/milestone/entrustment anchor (tagged or coded)<br>• % learners with a progression-mapped learning plan/ILP updated at least once per term with linked evidence (feedback/assessment artifacts)<br>• frequency of documented feedback-seeking events per learner per term (log or portfolio entries)<br>• time-to-first documented off-trajectory flag

		(days/weeks from start of term/rotation)<br>**Intermediate (transition to practice):**<br>• supervisor readiness rating at 4–8 weeks (1–5 or anchored scale)<br>• % at expected supervision/entrustment level at 4–8 weeks<br>• # documented early performance concerns flagged in first 8 weeks<br>• remediation/additional supervision rate during transition<br>• reduction in between-site variance in early readiness/entrustment ratings for comparable learners<br>**Distal (in practice):**<br>• proportion requiring enhanced supervision beyond expected period at 6–12 months<br>• early-career performance composite (6–12 months)<br>• rate of documented performance concerns/complaints in first year<br>• CPD/self-directed learning engagement (where available)
**Barriers / Enablers [49-51, 57, 84-91]**	**Barriers:**<br>• unclear/contested progression descriptors<br>• rater burden and inconsistent calibration; insufficient observation opportunities<br>• limited data infrastructure<br>• capacity constraints for regular checkpoint reviews<br>**Enablers:**<br>• shared anchors and calibration routines<br>• protected time for observation/coaching and review<br>• dashboards that visualize trajectory<br>• leadership support and clear decision rules<br>• faculty development for coaching to "next level"	• % assessments with direct observation<br>• observation opportunity distribution across sites<br>• calibration completion rate (%)<br>• median time-to-complete ratings<br>• % checkpoint reviews completed<br>• dashboard access rate and usage<br>• CQI action closure rate (%)
**Equity Considerations [31, 92, 93]**	Ensure progression expectations and decisions are equitable and that all learners have equitable access to observations, coaching, and opportunities needed to demonstrate progression (avoid opportunity gaps being misread as competence gaps).	• distribution of observation counts and feedback/coaching encounters by subgroup/site<br>• time-to-entrustment/progression thresholds by subgroup (with adjustment where feasible)<br>• differential rates of off-trajectory flags/remediation by subgroup<br>• narrative bias indicators in feedback<br>• equitable access to high-value experiences (opportunity index)<br>• proportion of equity flags triggering CQI actions and closed-loop completion (%)

CORE COMPONENT 3: TAILORED LEARNING EXPERIENCES

Core Component 3: Tailored Learning Experiences Elements	Description and Examples	Example Potential Indicators / Metrics
	Tailored learning experiences provide individualized, needs-driven opportunities that align with outcome competencies and progression (e.g., adapting experiences, coaching, assignments, case mix, or rotation selection based on learner needs and performance evidence) while ensuring required exposures and standards are met.[3]	• individualized learning pathway/plan process exists (Y/N)<br>• % learners with documented ILP/learning plan<br>• % learning experiences mapped to competencies and progression levels<br>• availability of flexible experiences/options (count of elective/targeted opportunities)
**Implementation Strategies [3, 22, 25, 33, 45, 49, 50, 57-60]**	• Use performance evidence (assessments, feedback, dashboards) to identify learning needs and tailor experiences.<br>• Implement ILPs/learning plans with specific competency-linked goals and actions.<br>• Create a menu of targeted learning supports (simulations, coaching sessions, skills labs, remediation resources, enrichment opportunities).<br>• Adjust rotations/case mix/assignments where feasible to address gaps (and prevent opportunity deficits).<br>• Ensure coaching/advising structures support tailoring and follow-up.<br>• Monitor equitable access to high-value learning opportunities and supports.	• % learners with ILP goals linked to competencies<br>• % ILPs updated each term with evidence; time from identified gap → targeted experience assigned (days)<br>• utilization rate of targeted supports (#/learner/term)<br>• % learners receiving tailored experiences matched to identified needs<br>• equity distribution of opportunities/supports by subgroup/site
**Drivers of Change [4, 5, 61]**	• One-size-fits-all curricula allow learners to progress with persistent gaps or without challenge.<br>• Variable clinical/experiential exposure produces opportunity gaps (what learners see/do differs widely).<br>• Supports/remediation are reactive, late, or inconsistent across sites.<br>• Learners lack structured mechanisms to translate assessment evidence into targeted learning actions.	• learners with unmet competency thresholds at key checkpoints<br>• variance in exposure/experience opportunities by site (opportunity index)<br>• time-to-intervention after concern (days)<br>• rate of late remediation initiated near transition<br>• learner report of ability to act on feedback (survey 1–5)
**Theory of Change [45, 46, 52] (Assumptions & Mechanisms)**	**Assumption:** learners vary in needs and pace; targeted experiences and supports improve the efficiency and equity of competency development.<br><br>**Mechanisms:** performance evidence identifies gaps → ILPs translate gaps into goals/actions → tailored experiences increase deliberate practice and appropriate exposure → coaching reinforces learning and monitoring → improved progression and reduced late-stage deficits.	• proportion of identified gaps that lead to a documented learning action<br>• time from gap identification → assigned targeted experience (days)<br>• improvement in targeted competency scores/entrustment levels after tailored experience<br>• reduced rate of persistent gaps across checkpoints
**Theoretical & Conceptual Foundations [62-80]**	• Mastery learning<br>• deliberate practice<br>• individualized instruction<br>• adaptive learning pathways<br>• self-regulated learning and coaching<br>• equity and opportunity-to-learn principles<br>• "assessment for learning" (using data to guide learning activities)	• presence of structured ILP template (Y/N)<br>• coaching fidelity (rubric-based)<br>• alignment of tailored activities to competency targets (audit)<br>• learner SRL behaviors (goal setting/monitoring frequency) captured in portfolios/logs
**Key Stakeholder Actions / Behavior Changes [81, 82]**	**Learners:**<br>• use evidence to identify gaps and create competency-linked goals<br>• engage in tailored activities<br>• reflect and update ILP based on new evidence<br>**Faculty/Preceptors:**	• % learners with ILP goals linked to competencies and updated each term<br>• completion rate of assigned tailored activities (%)<br>• # coaching touchpoints per learner per term

	• provide actionable feedback tied to competencies<br>• recommend targeted experiences; provide coaching and observe progress<br>• ensure opportunity-to-learn through planned exposure<br>**Administrators/Program Leaders:**<br>• ensure availability of targeted learning options and resources<br>• facilitate scheduling/placement flexibility<br>• monitor utilization and equity of access<br>• coordinate supports for learners off-trajectory	• % feedback entries that include an actionable “next step” and recommended learning activity<br>• time from need identification → support assigned (days)<br>• distribution of opportunities/support utilization by subgroup/site
**Implementation Outputs (Early Signals) [3, 15, 26, 27, 33, 45, 49, 50, 52, 53, 57, 69, 83]**	Early, observable signals that tailored learning experiences are implemented and functioning:<br>• ILP/learning plan process is established<br>• needs are identified using assessment evidence<br>• targeted learning options exist and are accessible<br>• assignments/experiences are adapted based on needs<br>• coaching/advising supports follow-up<br>• tracking systems document tailoring actions and participation	**Adoption/Reach:**<br>• % learners with ILP<br>• % faculty/preceptors trained in ILP/coaching<br>• availability of targeted options (count) and participation rate<br>**Uptake:**<br>• % identified needs linked to a documented tailored action<br>• utilization of targeted supports (#/learner/term)<br>**Fidelity/Consistency:**<br>• proportion of ILPs meeting quality criteria (specific, competency-linked, evidence-based)<br>• % coaching notes/action plans documented<br>• between-site variance in tailoring rates for comparable needs<br>**Feasibility:**<br>• time required to generate/update ILP<br>• scheduling success rate for targeted experiences<br>• acceptability score (1–5)<br>**Participation:**<br>• attendance at coaching sessions<br>• completion rate for tailored assignments/simulations
**Outcomes Over Time (Proximal, Intermediate, Distal) [3, 15, 26, 27, 33, 45, 49, 50, 52, 53, 57, 69, 83]**	**Proximal (in training):**<br>• increased learner engagement in goal-directed learning<br>• improved translation of feedback into targeted actions<br>• reduced opportunity gaps in observed experiences and coaching<br>**Intermediate (transition to practice):**<br>• fewer persistent competency gaps at graduation/transition<br>• reduced need for crisis remediation at transition<br>• smoother onboarding with fewer early performance concerns due to unaddressed gaps<br>**Distal (in practice):**<br>• sustained performance and continued professional development behaviors<br>• reduced early-career performance concerns attributable to unmet learning needs	**Proximal (in training):**<br>• % learners with ILP updated at least once per term with linked evidence<br>• frequency of documented goal-setting/monitoring/reflection entries<br>• % feedback entries linked to a specific learning action<br>• opportunity-to-learn index (exposure/observation counts) by site/subgroup<br>**Intermediate (transition to practice):**<br>• % meeting threshold on priority competencies/EPAs at transition<br>• supervisor readiness rating at 4–8 weeks (1–5)<br>• # early performance concerns in first 8 weeks<br>• remediation/additional supervision rate during transition<br>**Distal (in practice):**<br>• rate of documented performance

		concerns/complaints in first year<br>• CPD/self-directed learning engagement (portfolio activity or CME participation)<br>• performance composite at 6–12 months where feasible<br>• proportion requiring enhanced supervision beyond expected period at 6–12 months
**Barriers / Enablers [49-51, 57, 84-91]**	**Barriers:**<br>• limited flexibility in scheduling/placements<br>• insufficient targeted learning resources<br>• capacity constraints for coaching<br>• inconsistent use of assessment evidence to drive tailoring<br>• uneven exposure/case mix across sites.<br>**Enablers:**<br>• accessible dashboards/portfolios<br>• clear ILP templates and coaching routines; menu of targeted learning options<br>• leadership support for flexibility<br>• monitoring of opportunity equity.	• scheduling flexibility rate (% targeted placements achieved)<br>• # targeted learning options available and utilized<br>• coaching capacity (coach-to-learner ratio, # sessions/term)<br>• dashboard usage (# logins/month)<br>• between-site variance in exposure/opportunity index<br>• CQI action closure rate (%)
**Equity Considerations [31, 92, 93]**	Ensure tailored learning does not unintentionally widen gaps (e.g., some learners receiving fewer high-value opportunities) and that all learners have equitable access to experiences, observations, coaching, and supports needed to achieve outcome competencies.	• distribution of high-value experiences/observations/coaching encounters by subgroup/site<br>• utilization of targeted supports by subgroup (equity of access)<br>• time from need identification → support assignment by subgroup<br>• differential rates of unmet competency thresholds at checkpoints<br>• narrative feedback bias indicators<br>• proportion of equity flags triggering CQI actions and closed-loop completion (%)

CORE COMPONENT 4: COMPETENCY-FOCUSED INSTRUCTION

Core Component 4: Competency-Focused Instruction	Description and Examples	Example Potential Indicators / Metrics
	Competency-focused instruction ensures teaching, coaching, and supervision are intentionally designed to develop and assess outcome competencies, using evidence-informed methods (e.g., coaching, feedback, deliberate practice, direct observation, and scaffolded independence) aligned to progression expectations.[3]	• % sessions/rotations with competency-linked objectives<br>• % instructional activities mapped to competencies/progression levels<br>• availability of coaching/observation structures (Y/N)<br>• stakeholder familiarity with competency-focused instruction (survey 1–5)
**Implementation Strategies [3, 22, 25, 33, 45, 49, 50, 57-60]**	• Align instructional objectives and learning activities to competencies and progression levels (blueprinting).<br>• Train faculty/preceptors in coaching, direct observation, and competency-referenced feedback.<br>• Use structured opportunities for deliberate practice (simulation, cases, skills labs, repeated workplace observation).<br>• Embed formative assessment and feedback cycles (feed-forward).<br>• Use shared tools/anchors to reduce variability in instruction and supervision expectations.<br>• Provide resources and protected time to support observation and coaching.	• % faculty/preceptors completing coaching/feedback training<br>• # direct observation opportunities per learner per term<br>• % feedback documented within X days<br>• % learning activities explicitly competency-linked<br>• calibration participation rate (%)<br>• protected time adequacy (survey 1–5 or hours/term)
**Drivers of Change [4, 5, 61]**	• Instruction emphasizes content coverage over demonstrated performance.<br>• Limited direct observation and actionable feedback; coaching is inconsistent across sites.<br>• Learners receive vague feedback not linked to competencies or next steps.<br>• Opportunities for deliberate practice and scaffolded independence are uneven.	• % assessments based on direct observation<br>• feedback timeliness (% within X days)<br>• feedback specificity/actionability score (rubric-rated)<br>• between-site variance in observation counts/coaching encounters<br>• learner report of receiving actionable feedback (survey 1–5)
**Theory of Change [45, 46, 52] (Assumptions & Mechanisms)**	**Assumption:** competency development improves when instruction is aligned to explicit standards and supported by observation, coaching, and deliberate practice.<br><br>**Mechanisms:** competency-linked objectives → targeted instruction + deliberate practice → frequent direct observation → specific, actionable feedback → improved learner self-regulation and progression → readiness for transition and practice.	• increase in observation frequency<br>• improvement in feedback quality scores<br>• increase in competency-linked coaching notes<br>• improvement in targeted competency/entrustment ratings following coached practice cycles<br>• reduced persistent gaps across checkpoints
**Theoretical & Conceptual Foundations [62-80]**	• Deliberate practice<br>• mastery learning<br>• cognitive apprenticeship<br>• coaching and feedback theory<br>• workplace learning and entrustment<br>• formative assessment/assessment for learning<br>• scaffolding and progressive independence	• presence of structured coaching/observation tools (Y/N)<br>• coaching fidelity score (rubric-rated)<br>• proportion of teaching sessions using active learning/practice methods (%)<br>• faculty self-efficacy for coaching (survey 1–5).
**Key Stakeholder Actions / Behavior Changes [81, 82]**	**Learners**:<br>• engage in deliberate practice<br>• seek direct observation and feedback<br>• apply feedback and document reflection/next steps tied to competencies<br>**Faculty/Preceptors:**<br>• teach and supervise using competency-linked objectives	• # direct observation encounters per learner per term<br>• % feedback entries competency-linked and including actionable next steps<br>• feedback timeliness (% within X days)<br>• learner feedback-seeking frequency (#/term)

	• observe performance directly<br>• provide timely, actionable, competency-referenced feedback<br>• coach toward next-level behaviors<br>• adjust supervision based on readiness<br>**Administrators/Program Leaders:**<br>• ensure faculty/preceptor development and resources<br>• enable observation/coaching time<br>• standardize tools/expectations across sites<br>• monitor variability and implement CQI to strengthen instructional fidelity and equity	• % faculty/preceptors completing coaching training<br>• coaching contact frequency (#/learner/term)<br>• between-site variance in observation/coaching rates<br>• protected time adequacy (survey/hours)
**Implementation Outputs (Early Signals) [3, 15, 26, 27, 33, 45, 49, 50, 52, 53, 57, 69, 83]**	Early, observable signals that competency-focused instruction is implemented and functioning:<br>• competency-linked objectives and instructional plans are in place<br>• faculty/preceptors are trained in coaching and direct observation<br>• structured practice opportunities exist; observation and feedback cycles occur routinely<br>• supervision expectations are aligned to progression anchors; documentation systems capture coaching/feedback activity	**Adoption/Reach:**<br>• % courses/rotations using competency-linked objectives<br>• % faculty/preceptors trained<br>• tool usage (# coaching templates/entries)<br>**Uptake:**<br>• # observation events per learner per term<br>• % learners receiving feedback after observed performance<br>**Fidelity/Consistency:**<br>• feedback quality score (rubric-rated)<br>• % feedback competency-linked<br>• between-site variance in observation/coaching rates<br>• calibration concordance on supervision expectations<br>**Feasibility:**<br>• time to document feedback/coaching<br>• completion rates<br>• acceptability score (1–5)<br>**Participation:**<br>• attendance at coaching training/calibration<br>• sites participating in alignment checks
**Outcomes Over Time (Proximal, Intermediate, Distal) [3, 15, 26, 27, 33, 45, 49, 50, 52, 53, 57, 69, 83]**	**Proximal (in training):**<br>• increased frequency and quality of direct observation and coaching<br>• improved actionable feedback aligned to competencies/progression<br>• increased learner self-regulated learning behaviors (feedback seeking, reflection, deliberate practice)<br>**Intermediate (transition to practice):**<br>• improved readiness and supervision alignment<br>• fewer early performance concerns due to coaching gaps<br>• reduced need for crisis remediation at transition<br>**Distal (in practice):**<br>• sustained competent performance and continued professional development behaviors<br>• reduced early-career performance concerns potentially attributable to improved coaching/feedback during training	**Proximal (in training):**<br>• # direct observations per learner/term<br>• feedback quality score (specificity/actionability)<br>• % feedback linked to competency/progression anchor<br>• learner feedback-seeking events (#/term)<br>• reflection/learning plan updates with evidence (%)<br>**Intermediate (transition to practice):**<br>• supervisor readiness rating at 4–8 weeks (1–5)<br>• % at expected supervision/entrustment level at 4–8 weeks<br>• # early performance concerns flagged in first 8 weeks<br>• remediation/additional supervision rate during transition<br>**Distal (in practice):**<br>• rate of documented performance

		concerns/complaints in first year<br>• CPD/self-directed learning engagement (portfolio activity/CME)<br>• performance composite at 6–12 months where feasible<br>• proportion requiring enhanced supervision beyond expected period at 6–12 months
**Barriers / Enablers [49-51, 57, 84-91]**	**Barriers:**<br>• limited time for observation/coaching<br>• variable faculty/preceptor coaching skill<br>• high workload and competing mission demands<br>• inconsistent expectations across sites<br>• inadequate documentation tools<br>**Enablers:**<br>• protected time and staffing support<br>• faculty/preceptor development and calibration<br>• structured coaching/observation tools<br>• leadership expectations and accountability<br>• dashboards to monitor observation/feedback patterns	• protected time adequacy (survey/hours)<br>• training completion rate (%)<br>• coaching self-efficacy (survey 1–5)<br>• median time-to-feedback; observation rate variance across sites<br>• dashboard usage (# logins/month)<br>• CQI action closure rate (%)
**Equity Considerations [31, 92, 93]**	• Ensure all learners have equitable access to direct observation, coaching, feedback, and deliberate practice opportunities, and that instructional judgments and feedback language are bias-aware so opportunity gaps are not misread as competence gaps.<br>• Evaluate whether activities require resources that might result in variable ability to engage (e.g., requirements for technology, apps, references, access to labs, access to researchers, etc.)	• observation/coaching encounter distribution by subgroup/site<br>• feedback quantity and quality by subgroup (rubric-rated)<br>• time-to-observation/feedback by subgroup<br>• differential rates of early concerns/remediation by subgroup<br>• narrative feedback bias indicators (language audits)<br>• proportion of equity flags triggering CQI actions and closed-loop completion (%)

CORE COMPONENT 5: PROGRAMMATIC ASSESSMENT

**Core Component 5: Programmatic Assessment**	**Description and Examples**	**Example Potential Indicators / Metrics**
	Programmatic assessment is a coordinated system of multiple, low-stakes assessments collected over time and synthesized to support learning, progression, and high-stakes decisions about competence and readiness for practice. Decisions are based on patterns of evidence rather than single data points and are supported by governance, transparency, and quality assurance processes [3].	• existence of programmatic assessment framework/document (Y/N)<br>• % competencies/EPAs with multiple assessment sources<br>• presence of data synthesis and decision structures (Y/N)<br>• stakeholder understanding of programmatic assessment principles (survey 1–5)
**Implementation Strategies [3, 22, 25, 33, 45, 49, 50, 57-60]**	• Deploy multiple, low-stakes assessments aligned to competencies across settings and time.<br>• Separate formative assessment for learning from summative decisions, while ensuring alignment.<br>• Use portfolios/dashboards to aggregate evidence longitudinally.<br>• Establish committees (or equivalent governance structures) to synthesize evidence and make progression decisions using explicit decision rules.<br>• Train faculty/preceptors in assessment, feedback, and evidence interpretation.<br>• Embed CQI processes to monitor data quality, bias, burden, and decision consistency.	• # assessment tools per competency<br>• % assessments designated formative vs summative<br>• portfolio/dashboard availability (Y/N)<br>• % faculty/preceptors trained<br>• committee meeting frequency<br>• documented CQI cycles per year
**Drivers of Change [4, 5, 61]**	• Overreliance on single or episodic high-stakes assessments, which are insufficient to capture complex, context-dependent competence.<br>• Assessment systems that generate large volumes of data with limited interpretive value, due to inadequate sampling, inconsistent ratings, or lack of narrative feedback (i.e., low signal-to-noise).<br>• Fragmented assessment practices in which tools, feedback, and decisions are disconnected, limiting their usefulness for learning and progression.<br>• Inconsistent and opaque readiness decisions, driven by variable rater judgments and the absence of explicit synthesis and decision rules.<br>• Limited learner understanding of how assessment evidence is used, reducing engagement with feedback and undermining assessment for learning.<br>• Unmonitored bias and variability in assessment data that can distort judgments and exacerbate inequities.	• proportion of decisions based on single data points<br>• assessments per learner per term by competency<br>• proportion of assessments with narrative feedback<br>• missing data rate<br>• between-site variance in ratings for comparable learners<br>• learner/faculty understanding of assessment use (survey 1–5)<br>• appeals or contested decisions (%).
**Theory of Change [45, 46, 52] (Assumptions & Mechanisms)**	**Assumptions:**<br>• Competence is complex and context-dependent and is best judged through multiple observations over time; single assessments are insufficient.<br>• Programmatic assessment only functions as intended when it is intentionally designed with adequate sampling across contexts, appropriate tool–construct alignment, rater preparation, and transparent decision processes.<br><br>**Mechanisms:** effective assessment system design (aligned tools + sufficient sampling + rater calibration + low-stakes narrative feedback) → high-quality, longitudinal data (triangulated evidence) → structured synthesis using explicit decision rules → timely feedback and targeted supports → defensible progression/readiness decisions and improved learning and performance.	• assessment sampling adequacy (observations per learner per term<br>• coverage across settings/case mix)<br>• tool–competency blueprint completeness (% competencies with ≥2 data sources)<br>• rater training/calibration completion (%) and concordance<br>• data completeness (% required fields completed; missingness rate)<br>• proportion of assessments with narrative feedback and actionable next steps<br>• timeliness of feedback (median days)<br>• documentation completeness for synthesis/decisions (% with required elements)<br>• decision consistency across reviewers/committees (agreement rate)<br>• time from concern identification → support plan

		initiation (days)
**Theoretical & Conceptual Foundations [62-80]**	• Programmatic assessment as a system (multiple low-stakes data points over time)<br>• validity/interpretation argument logic (construct → assessment → inference → decision)<br>• purposeful sampling and triangulation/saturation across contexts<br>• assessment for learning (feedback loops and learner engagement)<br>• rater cognition and calibration<br>• decision-making under uncertainty (committee synthesis and rules)<br>• fairness, transparency, and equity in high-stakes decisions	• validity/interpretation framework documented (Y/N) and reviewed on schedule (Y/N)<br>• assessment sampling adequacy (observations per learner per term; coverage across settings/case mix)<br>• proportion of competencies/EPAs supported by ≥2 data sources (%)<br>• rater training/calibration completion (%) and concordance on benchmark cases<br>• assessment quality audit score (e.g., narrative specificity/actionability, linkage to competencies)<br>• documentation completeness for synthesis/decisions (% with required elements)<br>• decision agreement across reviewers/committees (agreement rate)<br>• missingness rate for required assessment fields (%)
**Key Stakeholder Actions / Behavior Changes [81, 82]**	**Learners**<br>• Engage with assessment as learning by seeking observation and feedback<br>• review longitudinal evidence (portfolio/dashboard) to identify patterns<br>• set competency-linked goals and action plans<br>• document reflection and follow-through<br>• use coaching/advising to adapt learning strategies based on evidence over time<br>**Faculty / Preceptors**<br>• Collect multiple direct observations across contexts<br>• provide timely, narrative, competency-referenced feedback with actionable next steps<br>• contribute evidence aligned to shared anchors rather than relying on single global ratings<br>• participate in calibration and feedback-quality improvement<br>• flag concerns early and document rationale/support plans.<br>**Administrators / Program Leaders** *(programmatic assessment design and stewardship)*<br>• Design and maintain a coherent program of assessment (blueprinting, tool selection, sampling plan, data integration, and decision rules)<br>• ensure transparency and learner access to evidence; resource rater training, calibration, and protected time<br>• establish governance for synthesis and defensible decisions<br>• monitor data quality, burden, bias, and consistency<br>• run closed-loop CQI to refine the system based on findings	• # assessments per learner per term (and coverage across contexts); % assessments based on direct observation<br>• % assessments with narrative feedback and actionable next steps<br>• feedback timeliness (median days)<br>• portfolio/dashboard engagement (logins/month; # reflection/action-plan entries/term)<br>• % competencies/EPAs supported by ≥2 data sources (triangulation)<br>• rater training and calibration completion (%) and concordance on benchmark cases<br>• decision documentation completeness (% with required elements) and decision-rule adherence (%)<br>• missingness rate for required fields<br>• assessment burden (median time-to-complete; acceptability score 1–5)<br>• equity/variability indicators (between-site variance; differential ratings/opportunities by subgroup)<br>• CQI closed-loop completion rate (% action items closed per cycle)
**Implementation Outputs (Early Signals) [3, 15, 26, 27, 33, 45, 49, 50, 52, 53, 57, 69, 83]**	Early, observable signals that programmatic assessment is implemented and functioning:<br>• assessment tools are deployed across settings<br>• sampling plans are enacted (sufficient observations across contexts)<br>• evidence is aggregated longitudinally (portfolio/dashboard)<br>• synthesis/review meetings occur<br>• decision rules are applied<br>• documentation is complete<br>• CQI processes monitor data quality, burden, and equity	**Adoption/Reach:**<br>• % courses/rotations contributing assessment data; % stakeholders trained<br>• dashboard usage (# logins/month)<br>**Uptake:**<br>• % learners with ≥X assessments per term<br>• % assessments submitted on time<br>• % competencies/EPAs supported by ≥2 data sources (triangulation)

<table>
<tr>
<td></td>
<td></td>
<td>Fidelity/Consistency:
calibration agreement on benchmark cases
between-site variance in ratings for comparable learners
% decisions adhering to documented rules
% assessments with narrative feedback and actionable next steps
Feasibility:
median time to complete assessments
completion rates
acceptability score (1–5)
Participation:
attendance at synthesis/decision meetings
% sites participating in data review cycles
CQI closed-loop completion rate (% action items closed per cycle)</td>
</tr>
<tr>
<td>Outcomes Over Time (Proximal, Intermediate, Distal) [3, 15, 26, 27, 33, 45, 49, 50, 52, 53, 57, 69, 83]</td>
<td>Proximal (in training):
richer feedback
improved learner understanding of expectations
earlier identification of performance patterns
increased learner engagement with assessment data.
Intermediate (transition to practice):
more defensible readiness decisions
fewer late-stage “surprises”
smoother transition with supervision aligned to evidence
Distal (in practice):
sustained competent performance
trust in assessment-based readiness decisions
reduced early-career performance issues</td>
<td>Proximal (in training):
feedback quality score using a structured rubric (specificity/actionability/competency linkage)
% assessments with narrative feedback and actionable next steps
% learners reporting understanding of how assessment evidence informs decisions (survey 1–5) and/or able to state current performance pattern + next steps (brief prompt scored Y/N)
time-to-first documented concern/pattern flag (days/weeks)
portfolio/dashboard engagement (logins/month; # reflection/action-plan entries/term)
completeness of required evidence fields (missingness rate %)
Intermediate (transition to practice):
supervisor readiness rating at 4–8 weeks (1–5 or anchored scale)
% at expected supervision/entrustment level at 4–8 weeks
# documented early performance concerns in first 8 weeks
remediation/additional supervision initiation rate during transition
documentation completeness of readiness decisions (% with required elements) and/or decision-rule adherence rate (%)
Distal (in practice):
rate of performance concerns/complaints in first year
employer/supervisor trust score (1–5)
early-career performance composite at 6–12 months
proportion requiring enhanced supervision beyond expected period</td>
</tr>
</table>

		• practice-pattern quality/safety indicators or resource utilization measures
**Barriers / Enablers [49-51, 57, 84-91]**	**Barriers:**<br>• assessment burden<br>• insufficient faculty time/expertise<br>• poor data infrastructure<br>• resistance to narrative assessment<br>• unclear governance<br>**Enablers:**<br>• leadership ownership<br>• faculty development<br>• efficient tools/dashboards<br>• clear decision rules<br>• protected time for synthesis<br>• routine CQI	• assessment completion timeliness<br>• faculty workload estimates<br>• IT readiness score<br>• training completion rate (%)<br>• frequency of synthesis meetings<br>• CQI action closure rate (%)
**Equity Considerations [31, 92, 93]**	• Ensure assessment practices, synthesis, and decisions are fair, bias-aware, and transparent, and that opportunity gaps are not misinterpreted as competence gaps.	• Assessment volume and quality by subgroup/site<br>• narrative language bias audits<br>• differential rates of concern flags/remediation by subgroup<br>• appeals/review outcomes by subgroup<br>• equity flags triggering CQI actions and closed-loop completion (%)
